# A hybrid framework for enhanced segmentation and classification of colorectal cancer histopathology

**DOI:** 10.3389/frai.2025.1647074

**Published:** 2025-10-14

**Authors:** Aaseegha M. D., Venkataramana B.

**Affiliations:** Department of Mathematics, School of Advanced Sciences, Vellore Institute of Technology, Vellore, India

**Keywords:** colorectal cancer (CRC) diagnosis, ResUNet-A, EfficientNet, Swin Transformer, self-attention in transformers

## Abstract

**Introduction:**

Colorectal cancer (CRC) remains one of the leading causes of cancer-related deaths globally. Early detection and precise diagnosis are crucial in improving patient outcomes. Traditional histological evaluation through manual inspection of stained tissue slides is time-consuming, prone to observer variability, and susceptible to inconsistent diagnoses.

**Methods:**

To address these challenges, we propose a hybrid deep learning system combining Swin Transformer, EfficientNet, and ResUNet-A. This model integrates self-attention, compound scaling, and residual learning to enhance feature extraction, global context modeling, and spatial categorization. The model was trained and evaluated using a histopathological dataset that included serrated adenoma, polyps, adenocarcinoma, high-grade and low-grade intraepithelial neoplasia, and normal tissues.

**Results:**

Our hybrid model achieved impressive results, with 93% accuracy, 92% precision, 93% recall, and 93% F1-score. It outperformed individual architectures in both segmentation and classification tasks. Expert annotations and segmentation masks closely matched, demonstrating the model’s reliability.

**Discussion:**

The proposed hybrid design proves to be a robust tool for the automated analysis of histopathological features in CRC, showing significant promise for improving diagnostic accuracy and efficiency in clinical settings.

## 1 Introduction

Colorectal cancer (CRC) is one of the most frequent cancers and a major cause of cancer-related death globally, albeit it is not the most common cause of death in general (Sirinukunwattana et al., 2021). Age, lifestyle changes, and genetic susceptibility are some of the factors contributing to the increased prevalence of colorectal cancer (CRC), according to WHO (World Health Organisation, 2025) and GLOBOCAN statistics (Sung et al., 2021). Improving survival rates requires early discovery and precise diagnosis. Histological evaluation of biopsy tissue has long been the gold standard. However, this procedure is subjective, time-consuming, and vulnerable to fluctuation among observers (Tamang and Kim, 2021). Therefore, the hardneed for automated, objective, and effective computational methods to support pathologists in segmentation and classification tasks is growing (Ponzio et al., 2018).

Artificial intelligence (AI) advances in the last few years, especially Deep learning (DL), have made a tremendous difference in medical image analysis by providing highly accurate, automated disease detection, segmentation and classification methods. DL frameworks, particularly Convolutional Neural Networks (CNNs), have proven incredibly useful in processing histopathological photos due to their ability to automatically extract important elements from unprocessed image data (Karthikeyan et al., 2024). Contrary to traditional machine learning techniques, where feature extraction is typically done manually, CNNs automatically learn hierarchical features in histopathological images, enabling better generalized and more stable pattern identification (Paladini et al., 2021).

Several DL architectures have been suggested as a way to deal with classification issues in medical imaging (MI). Among the most popular architectural designs in medical image segmentation, U-Net, employs an encoder-decoder model for feature extraction, utilizing skip connections to preserve spatial information (Sun et al., 2019). While U-Net is effective at segmenting biological images, it struggles with fine-grained discrimination of tissues due to its reliance on local feature extraction (Raju et al., 2025a). ResNet, a popular deep network, uses residual learning to address vanishing gradients, improving feature extraction, segmentation, and classification. However, standalone CNN models lack global context, important for distinguishing similar colorectal cancer tissues. To overcome this, hybrid deep learning frameworks combine different architectures to leverage their strengths (Selvaraj et al., 2025).

Combining attention mechanisms and transformers has emerged as a highly effective approach in medical imaging. Vision Transformers (ViTs) have been particularly effective in MI (medical image) processing since they can pick up long-range relationships (Ayana et al., 2024). ViTs utilize self-attention mechanisms to understand both local and global picture features, which results in more accurate classification than CNNs based on local receptive fields (Zeid et al., 2021). The Swin Transformer has garnered considerable attention for its window-shifting mechanism, which enhances processing efficiency while preserving spatial hierarchies (Zidan et al., 2023). Combining transformers with CNN-based architectures allows DL models to achieve better segmentation and classification performance, making them highly efficient in processing histopathology images. Each of the architectures is capable of boosting overall model performance separately. ResUNet-A, being an extension of U-Net, combines residual connections and attention mechanisms to enhance feature propagation and emphasize significant histological features (Ahamed et al., 2024). Residual learning improves gradient propagation, enabling deeper network training and more precise segmentation by focusing on significant tissue areas. EfficientNet, with its compound scaling strategy, efficiently extracts complex histopathological features by balancing depth, width, and resolution (Girepunje and Singh, 2024). The Swin Transformer, through self-attention and shifted windowing, captures long-range dependencies and global context, making it more effective for high-resolution histopathology images (Dutta et al., 2024).

Our proposed model differs from conventional CNN-Transformer hybrids, which typically combine only a single CNN backbone with a Transformer, by integrating ResUNet-A, EfficientNet, and Swin Transformer. We proposed a hybrid deep learning model that combines residual learning, efficient feature extraction, and self-attention. This integration enhances segmentation accuracy, optimizes feature extraction, and improves global context modelling, thereby reducing misclassification risks. Our model outperforms existing approaches, such as U-Net, ResNet, and conventional ResUNet-A, by leveraging the complementary strengths of these three architectures for colorectal histopathology images. The novelty of this study lies in developing a unified hybrid framework for colorectal cancer histopathological image analysis, which has not been explored in prior CRC studies. The primary contributions include: (i) improved segmentation precision and boundary delineation with ResUNet-A, (ii) optimized feature extraction, stable tissue classification and recognition of complex histopathological patterns using EfficientNet, and (iii) enhanced global context and long-range dependency modeling through the Swin Transformer’s self-attention. Collectively, these ensure higher diagnostic reliability with minimal human oversight.

The manuscript is organized as follows: Section 2 reviews related deep learning methods for cancer diagnosis and segmentation; Section 3 outlines the background of ResUNet-A, EfficientNet, and Swin Transformer; Section 4 presents the proposed hybrid model, data augmentation, training, and evaluation; Section 5 discusses experimental results, comparisons, and statistical validations; and Section 6 concludes with key contributions and deployment prospects.

## 2 Literature review

See Table 1.

**Table 1:** Summary of classification and segmentation techniques across various fields utilizing deep learning methods.

**Ref**	Domain	Model	Advantages strengths	Limitations/Gaps	Dataset	Reported Performance
Dutta et al. (2024)	Brain tumor classification	ARM-Net (multiscale CNN) + RM-Net with LGAM (lightweight global attention)	Captures multi-scale features; LGAM selectively emphasizes discriminative traits; lightweight attention.	Potential dataset specificity; attention adds complexity; simplified residual blocks may lose fine detail.	Brain tumor datasets (not specified).	Not explicitly reported.
Cheng et al. (2022)	Medical image segmentation/classification backbone	ResGANet (ResNet-like with modular group attention blocks)	1.51–3.47 × fewer parameters than ResNet; strong downstream segmentation; efficient attention.	Parameter reduction may limit representational capacity; requires validation across diverse modalities.	General medical images; stacked ResGANet.	Fewer params; exact scores not provided.
Fan et al. (2023)	Remote sensing land-use categorization	MARC-Net: multi-scale residual cascade + multi-head attention (parallel framework)	Model’s spatial interrelationships; mines spectral embeddings; robust multi-scale representation.	Domain-specific; increased model complexity; limited clinical relevance.	Remote sensing images.	Not explicitly reported.
Li et al. (2022)	Medical image segmentation (coronary angiography, nuclei, skin cancer)	Residual-Attention UNet++	Residual units mitigate degradation; attention suppresses irrelevant background to focus on targets.	Higher computational cost; potential overfitting if data are limited.	Multiple medical datasets (angiography, nuclei, skin).	Qualitative improvement; no single numeric summary stated.
Zhang et al. (2020)	Brain tumor segmentation (2D)	AResU-Net (Attention Residual U-Net)	Combines residual learning with attention for improved focus on tumor regions.	2D only (misses 3D context); requires large labeled data; compute overhead from attention.	2D MRI brain tumor datasets.	Not explicitly reported.
Wang et al. (2020)	Retinal fundus multi-label abnormality detection	Ensemble CNN with EfficientNet feature extractor + custom classifier	Direct multi-label prediction; strong transfer learning via EfficientNet.	Sensitive to class imbalance; needs extensive data curation and calibration for multi-label thresholds.	Fundus photo datasets.	Not explicitly reported.
Alhichri et al. (2021)	Remote sensing scene classification	CNN with deep attention (EfficientNet-B3-Attn-2)	Attention reweights salient features; leverages strong EfficientNet backbone.	Relies on pretraining; domain shift may reduce performance.	Remote sensing scenes.	Not explicitly reported.
Oyediran et al. (2024)	Lung cancer detection (CT)	Chameleon Swarm Algorithm (CSA) optimized SVM; preprocessing + FCM segmentation + LBP features	CSA tuning boosts accuracy; reduced false positives; efficient classical pipeline.	Hand-crafted features may not generalize; multi-stage preprocessing; limited to CT modality.	CT scans (normal/benign/malignant).	Avg recognition accuracy 95.64%; improved sensitivity & specificity.
Raza et al. (2023b)	Remote sensing scene classification	Deep attention CNN (EfficientNet-B3-Attn-2)	Saliency-aware feature maps; improved representation over plain CNN.	Similar limitations as potential redundancy with related works.	Remote sensing scenes.	Not explicitly reported.
Atila et al. (2021)	Plant leaf disease classification	EfficientNet (transfer learning) vs. other DL models	Strong performance with transfer learning, a large curated dataset, and efficient scaling.	PlantVillage’s lab-like images’ generalisation to field conditions may drop.	PlantVillage: 55,448 original / 61,486 augmented images.	Comparative study; exact best scores vary by model.
Huang et al. (2022)	Ship detection & classification	CNN-Swin (parallel CNN + Transformer with self-attention); CNN block to prevent overfitting	Captures multi-scale/global context; transformer improves discrimination.	Computation and memory cost; dataset is military-ship specific.	FGSC-23 military ship dataset.	Not explicitly reported.
Iqbal and Sharif, 2023)	Breast tumor classification & segmentation	BTS-ST network: U-Net with Swin Transformer; SIBs and FCBs	Global modeling via Swin; SIBs improve spatial correlation; FCBs help small-tumor segmentation.	Transformer modules are resource-intensive; patch tokenization may still lose detail.	Breast tumor datasets (not specified).	Not explicitly reported.
Zhang et al. (2021)	Rice disease identification (field images)	Hierarchical Swin-Transformer with sliding window	High accuracy in field settings; strong global/local feature fusion.	Accuracy 93.4%—room for improvement; performance may vary with lighting and occlusion.	Field-captured rice disease images.	93.4% accuracy (beats classical ML baseline ~4.1%).
Chen et al. (2022)	Lung cancer cell detection (microscopy)	CNN + Swin Transformer; Mask R-CNN pre-segmentation; Gaussian blur context handling	Outperforms ResNet-50; reduces compute vs. heavy CNNs; isolates cells before classification.	Multi-stage pipeline complexity; dependence on accurate instance segmentation.	Microscopic lung cell images.	Qualitatively superior; numbers not specified.
Murugesan et al. (2023)	Colorectal cancer stage identification (colonoscopy)	YOLOv3-MSF with ResUNet-based anchors + K-Medoids; FC layer for staging	Multi-scale detection; integrates polyp dimensions; strong metrics vs. Faster-RCNN.	Relies on accurate polyp segmentation/measurement; needs clinical validation.	CVC-ColonDB (1,000 HD images; 500 early malignant / 500 non-cancer).	Accuracy 96.04%; high precision/recall/F1.
Cao et al. (2024)	Gastric precancer segmentation in MHSI	MT-SCnet (Transformer): Multi-Scale Token Division + SCFormer + deformable conv	Excellent Dice/IoU; good global contextual fusion; reduced semantic gaps; lower compute vs. SOTA.	Requires hyperspectral imaging hardware; training complexity.	Two gastric MHSI datasets (GIN, IM).	Outperforms SOTA in accuracy, sensitivity, IoU, Dice.

## 3 Background study

### 3.1 Residual U-Net

**Figure 1:**
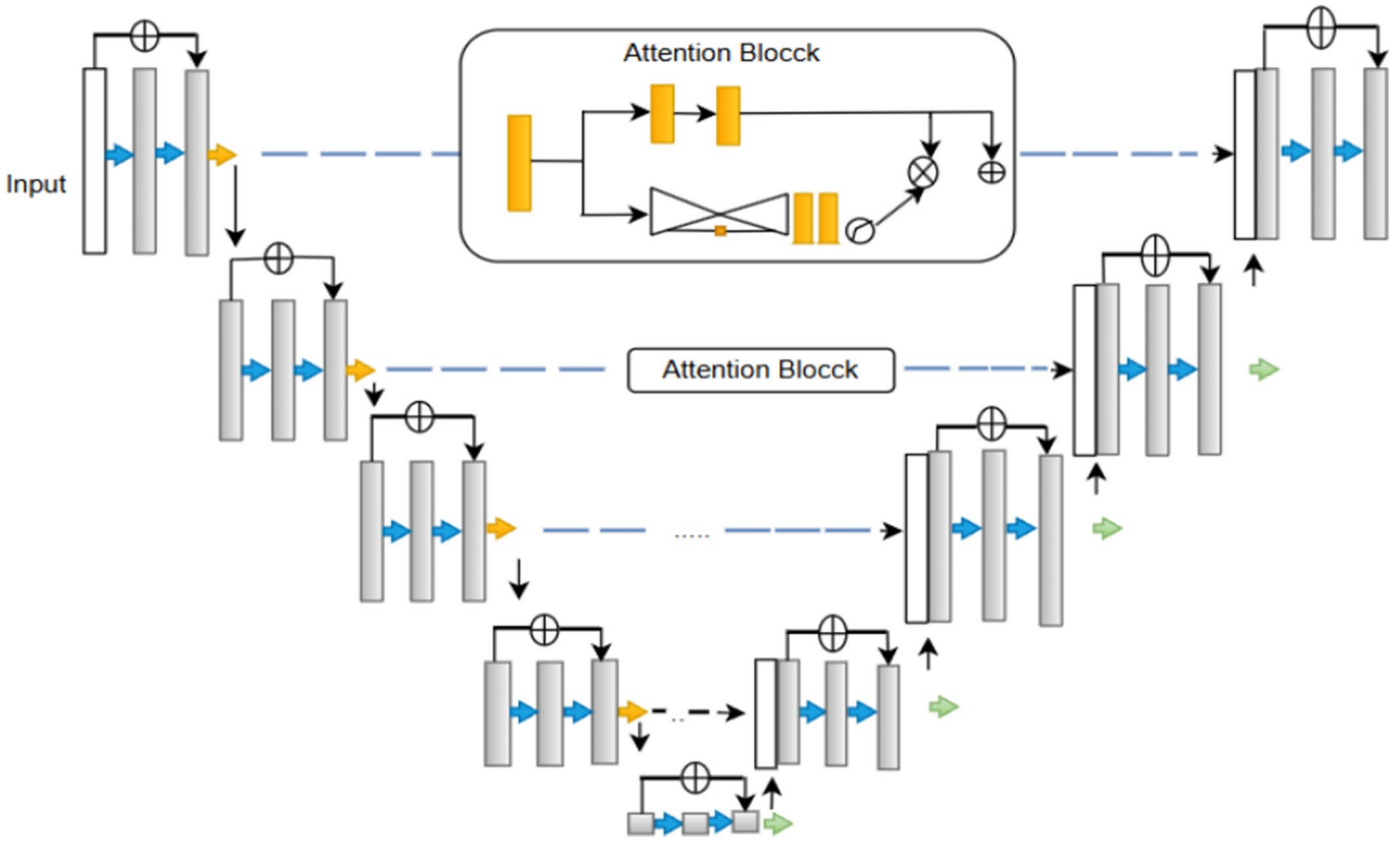
Structure of residual U-Net.

U-Net is a “U-shaped” Convolutional neural network architecture used for segmentation. As shown in Figure 1. The U-Net consists of two primary components: the encoder and the decoder. The encoder extracts the high-resolution input image’s features, and the decoder produces the final output, which also upsamples intermediate features. There are pathways connecting the symmetrical encoder and decoder (Ronneberger et al., 2015). The vanishing gradient problem plagues neural network training. Backpropagation is used to calculate the gradient, which is the derivative of the loss function concerning the weights, to update the weights. At the network’s earlier levels, the gradient becomes incredibly small. When the gradient is vanishingly small, the weights update proportionally to it and change only slightly. The weights consequently become trapped and never update to their ideal value. As such, it hinders the network’s ability to learn.

The Residual U-Net primarily addresses this problem by incorporating residual blocks into the U-Net architecture (Alwan et al., 2024). A collection of layers with a shortcut connection that bypasses one or more levels is referred to as a residual block (Equation 1) (Raza et al., 2023a). Provides the block’s output.


(1) $$y=F(x,\{W_{i}\})+x$$

-
x = input to the residual block.
-
y = output of the residual block.
-
$W_{i}$ = weights of the i^th^ layer in the residual unit.
-
$F(x,\{W_{i}\})\;$ = transformation function (e.g., a sequence of convolutional, batch normalization, and activation layers) applied to the input x .

The function of the residual block is usually a sequence of convolutional layers. Since this topology has shortcut connections, gradients can travel directly through the network’s layers. While training deeper networks, convergence is faster and more stable.

### 3.2 EfficientNet

In deep learning, the EfficientNet architecture has revolutionized the field, particularly for tasks such as image object recognition (Tan and Le, 2019). EfficientNet-B0, one of its primary variants, has drawn considerable interest due to its effective performance and efficient resource usage in image classification. It is well known for its compound scaling technique, which modifies the model’s complexity and size to strike the ideal balance. This makes it suitable for AI models with limited computing power, as it provides good accuracy. The EfficientNet model was initialized in this investigation using noisy student weights. This enhances the model’s functionality and task-specific adaptability.

EfficientNet-B0 utilizes a network architecture for scalable image classification. EfficientNetB0, the foundational model of the EfficientNet family, uses a compound scaling mechanism that scales the network’s depth, width, and resolution in equal proportions. The model design enables both high-accuracy performance and effective operation. The numerous components of the network design, ranging from Module 1 through Module 584, Alruwaili and Mohamed (2025) are referred to as modularity. Individual network components, including pooling layers and convolutional or activation layers, are represented by the modular method in network architecture. EfficientNetB0’s systematic and hierarchical organization, along with its modules 1–584, optimizes performance and resource use.

Compound scaling, which proportionally alters the network’s depth, width, and input resolution, is used to build EfficientNet-B2 from the fundamental EfficientNet-B0 model. In a more detailed analysis, B2 exhibits a greater model depth, more channels in the convolutional block, and a higher input picture resolution (260 × 260) compared to B0. The improvements made to EfficientNet-B2 enable it to encode finer-scale spatial patterns more effectively (Tan and Le, 2021). Compared to B0, EfficientNet-B2 contains about 9.1 million less parameters. However, its somewhat higher accuracy makes it a good option for tasks requiring finer information, such medical picture classification and fine-grained categorization. B2’s layout design and dimension scaling allow it to scale while preserving computing performance.

**Figure 2:**
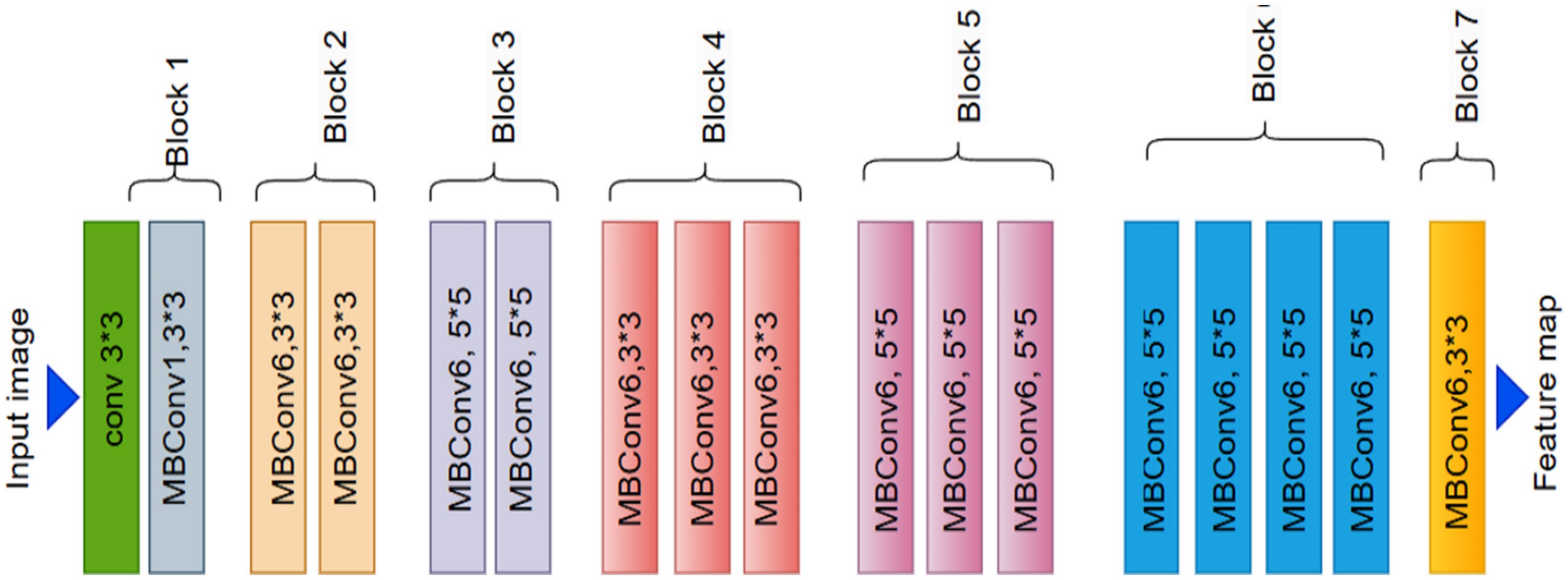
EfficientNet architecture.

EfficientNetB2 is a scalable convolutional neural network for image categorization. As shown in Figure 2. The several numbered modules, from Module 1 to 586, demonstrate a modular design structure. Each module corresponds to a distinct network component, including pooling blocks, activation functions, and convolutional modules. The order of these modules illustrates how EfficientNetB2 follows a hierarchical approach to maximize resource efficiency and deep learning performance (Tan et al., 2020). To increase model accuracy and robustness, EfficientNetB0 upgrades leverage extended scaling parameters.

### 3.3 Swin transformer

Convolutional Neural Networks struggle to represent connections and global context within an image. Here, ViT can play a helpful role in addressing this problem (Angona and Mondal, 2025a). Vision transformers excel in visual tasks, leveraging self-attention mechanisms to capture long-range correlations in raw data, surpassing CNNs’ performance. Nevertheless, vision transformers face challenges with high-resolution pictures. Swin transformers expand on the ViT model’s success to address this issue (Dosovitskiy et al., 2021). In 2021, the swing transformer architecture was initially presented (Liu et al., 2021). They perform better because swing transformers can process huge images with less computational complexity than vision transformers.

**Figure 3:**
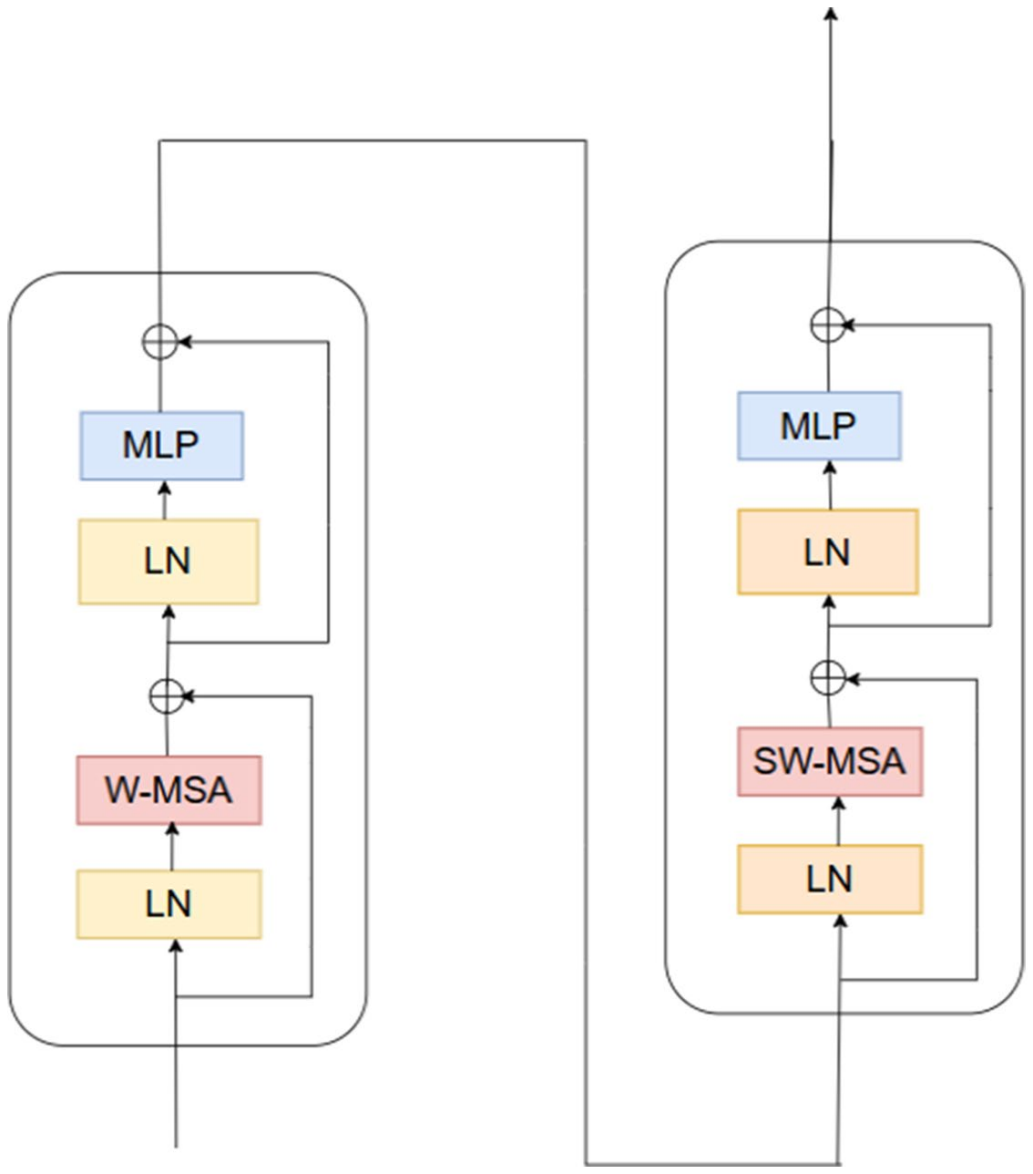
Swin Transformer architecture.

Pacification is the initial stage in the Swin transformer architecture. The initial step involves segmenting the input image into distinct patches. The transformation of image pixels into a vector or numerical representation is achieved through the application of linear embedding layers. The transformer blocks are then fed these vectors. Figure 3 shows the structure of a Swin transformer block. Subunits make up swing transformer blocks. A multi-layer perceptron layer, another normalizing layer, an attention layer, and a normalization layer make up each subunit. The W-MSA (window multi-head self-attention) in the first subunit computes attention within non-overlapping windows. Focusing the self-attention computation on local regions lowers the computational complexity. It is explained how computationally complex MSA and W-MSA are in Equations 2, 3. Whereas W-MSA scales linearly with the number of patches, MSA scales quadratically.


(2) $$\Omega (\mathrm{MSA})=4\mathrm{hw}C^{2}+2{\left(\mathrm{hw}\right)}^{2}C$$

(3) $$\Omega (W-\mathrm{MSA})=4\mathrm{hw}C^{2}+2M^{2}\mathrm{hwC}$$

-
h,w : height and width of the input feature map.
-
C : number of feature channels.
-
M : window size in the Swin Transformer.
-
Ω(MSA): computational complexity of global Multi-Head Self-Attention.
-
Ω(W−MSA) : computational complexity of Window-based Multi-Head Self-Attention.
-
$4\mathrm{hw}C^{2}$ : cost of linear projections (query, key, value, and output).
-
$2{\left(\mathrm{hw}\right)}^{2}C$ : attention cost for global MSA, quadratic with respect to spatial size.
-
$2M^{2}\mathrm{hwC}\;$ : attention cost for W-MSA, linear with respect to window size.

For high-resolution images, in particular, $\;{\left(\mathrm{hw}\right)}^{2}C$ , the quadratic term of the original MSA gets expensive with an increasing quantity of hw patches. When compared, the scalability of the linear term in Ω(W−MSA) , $2M^{2}\mathrm{hwC}$ , is significantly greater. The term 2 M remains constant as the number of patches w increases because the window size M is constant. The second subunit uses SW-MSA (shifted window multi-head self-attention). In this case, the cross-window linkages are shown using the cycle shift approach, which aids the model in capturing the global context. The implementation of self-attention occurs within the context of shifted windows, which are arranged according to a cyclic shift mechanism. The self-attention mechanism of the Swin Transformer utilizes a relative positional bias, which significantly improves the ability to capture positional correlations among patches. The given Equation 4 defines the attention function (Pacal et al., 2025).


(4) $$\text{Attention}(Q,K,V)=\text{Softmax}({\mathrm{QK}}^{T}/\sqrt{d}+B)$$

- Q = Query matrix
- K = Key matrix
- V = Value matrix
- d = dimensionality of query/key vectors
- B = relative positional bias

To calculate attention weights, queries (Q), keys (K), and values (V) are extracted from input patches; d is a vector dimension, and B is denoted by relational positional bias matrix that takes into consideration the positional relationships between patches (Rajasekar et al., 2024). Two fully connected layers with non-linear activation make up the multi-layer perceptron layer. Non-linear interactions between features are captured in this way. Before and after each MSA and MLP layer, layer normalisation improves training stability. The input patches are integrated within the output through the establishment of a residual link, thereby avoiding the need to traverse the entire block. This helps to preserve information and avoid fading gradients. Finally, the Swin transformer efficiently captures global information by selecting and merging the nearby patches. Patch merging is a hierarchical process that downsamples the image by a factor of N and concatenates M neighbouring patches along the channel dimension (Angona and Mondal, 2025b).

**Figure 4:**
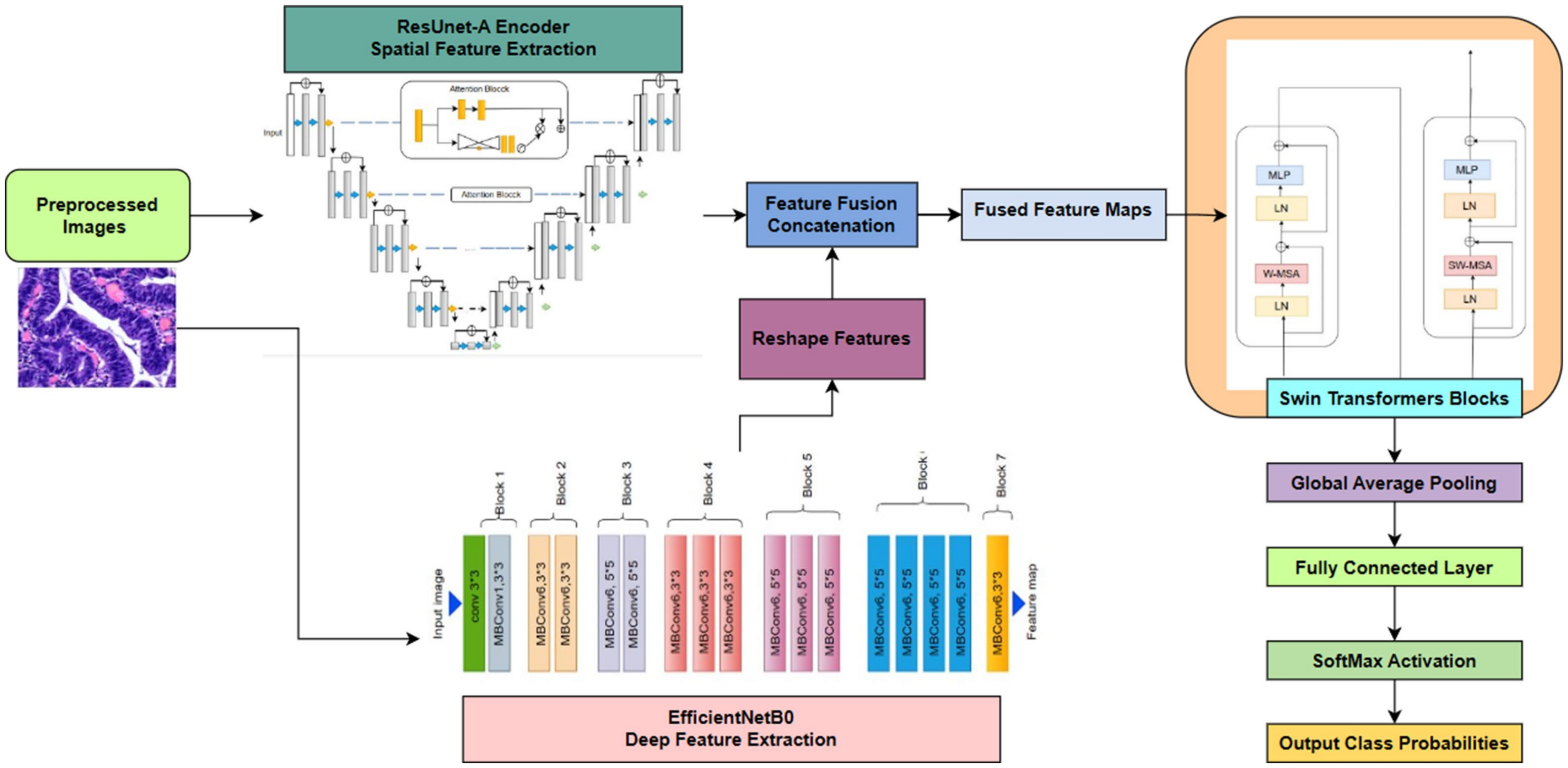
Proposed framework.

This suggested approach is an advanced hybrid deep learning architecture in Figure 4 intended for intricate picture analysis jobs. It utilizes the synergistic strengths of three robust models: a ResUNet-A Encoder to maintain accurate spatial details and multi-scale features, EfficientNetB0 for the efficient extraction of deep, hierarchical semantic features, and a Swin Transformer to capture long-range contextual dependencies and global relationships within the integrated feature set. By amalgamating multiple parallel feature extraction paths and augmenting the integrated features with transformer-based contextual reasoning, the model generates a robust and comprehensive representation of the input image. This synergistic approach, enhanced by Adam and regularised to prevent overfitting, is particularly useful for situations requiring both precise localisation and a comprehensive understanding of visual context, such as medical imaging or detailed classification.

## 4 Methodology

### 4.1 Data collection

The dataset used in this study is the publicly available Enteroscope Biopsy Histopathological Hematoxylin and Eosin Image Dataset for Image Segmentation Tasks (EBHI-Seg), comprising 4,456 histopathology images, including 2,228 raw histopathological section images and 2,228 corresponding ground truth images. The dataset was prepared by 12 biomedical researchers and validated by two histopathologists at the Cancer Hospital of China Medical University (Ethics certification no. 202229). Each image was assigned a label according to the most prominent differentiation stage present, with the most severe and clearly visible stage used when multiple differentiation stages appeared in a single image (Shi et al., 2023). The dataset is publicly available and can be accessed from MIaMIA Group (2022).

**Figure 5:**
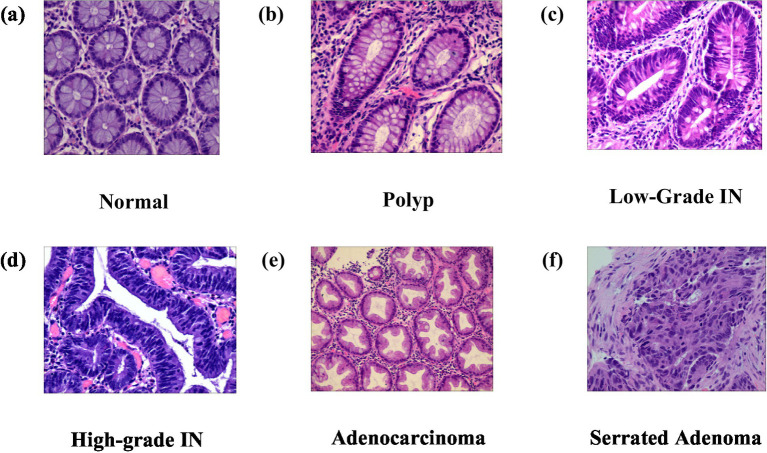
**(a–f)** Represent adenocarcinoma, high-grade IN, low-grade IN, normal, polyp, serrated adenoma.

The Enteroscope Biopsy Histopathological Haematoxylin and Eosin (H&E) Image Dataset for Image Segmentation Tasks (EBHI-Seg) was developed utilising intestinal biopsy specimens. Images were obtained at a magnification of 400 × (10 × eyepiece and 40 × objective) using a Nissan Olympus microscope and NewUsbCamera acquisition software. All images were stored in RGB format (.png) with a standardised size of 224 × 224 pixels, rendering them appropriate for segmentation and classification tasks. In Table 2 The dataset includes six tissue categories: In Figure 5a Normal (well-ordered tubular colorectal tissue without infection), Figure 5b Polyp (benign mucosal overgrowth with intact luminal structures and minimal nuclear division), Figure 5c Low-Grade Intraepithelial Neoplasia (Low-Grade IN) (precancerous lesions characterised by increased branching, dense arrangements, and mildly enlarged nuclei), Figure 5d High-Grade Intraepithelial Neoplasia (High-Grade IN) (advanced precancerous lesions exhibiting significant structural abnormalities and frequent nuclear division), Figure 5e Adenocarcinoma (malignant colorectal tumours with irregular luminal structures and markedly enlarged nuclei), and Figure 5f Serrated Adenoma (rare lesions, constituting approximately 1% of colonic polyps, histologically similar to colonic adenomas). The variety of tissue categories establishes EBHI-Seg as a reliable benchmark for the development and assessment of histopathology image segmentation and classification algorithms. All images in EBHI-Seg are organized into subdirectories for efficient loading and labeling, ensuring reproducibility and scalability. The dataset includes both benign and malignant samples, providing variability and balanced class representation for colorectal cancer segmentation and classification tasks.

**Table 2:** Class distribution table.

Class	Number of Images
Normal	76
Polyp	474
Low-Grade IN	639
High-Grade IN	186
Adenocarcinoma	795
Serrated Adenoma	58
Total	2,228

### 4.2 Data preprocessing

**Figure 6:**
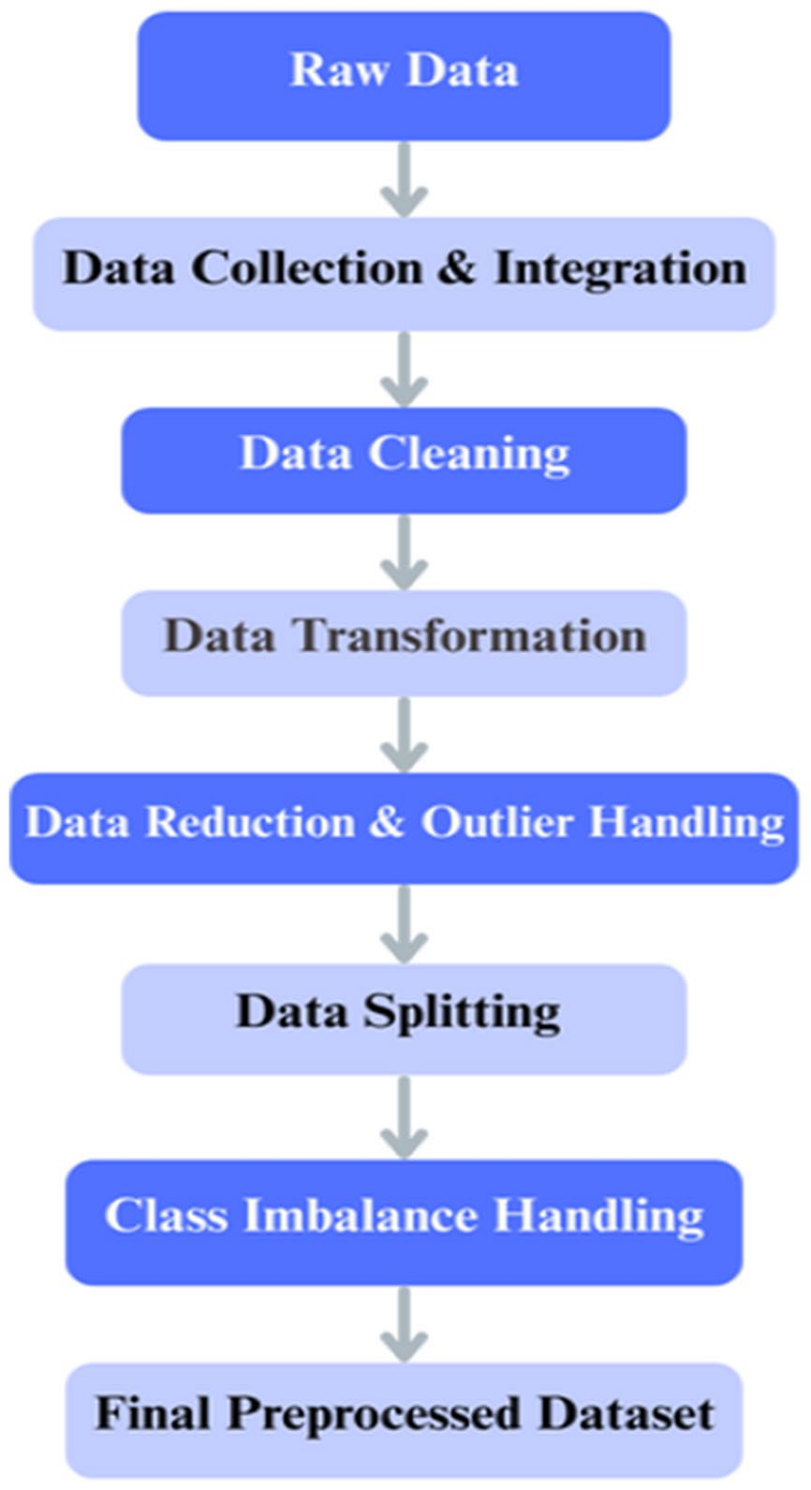
Overall step-by-step procedure illustrating the preprocessing workflow.

Data preparation is an essential process during the application of DL methods for histopathology image categorization in colorectal cancer (Kim et al., 2023). The data preprocessing pipeline ensured the quality and consistency of the input data before model development, as illustrated in Figure 6. After collection and integration, data cleaning was performed to resolve missing values and inconsistencies, followed by normalization, feature encoding, dimensionality reduction, and outlier handling. The dataset was then split into training, validation, and test sets, with class imbalance addressed only in the training set through oversampling and a class-weighted loss function. Images were resized and augmented using zooming, shearing, shifting, rotations, and horizontal flipping to improve generalization.

#### 4.2.1 Image resizing and normalization

To maintain morphological details while ensuring computational efficiency, the histopathology images were set to a resolution of 128×128 pixels (Deng et al., 2009). Resizing maintains essential attributes for categorization while lowering processing demands. To stabilize gradient computations during backpropagation, pixel intensity data were normalized to the [0, 1] range by dividing by 255 (LeCun et al., 2015).

#### 4.2.2 Label encoding

Using integer mappings, the categorical labels of the tissue types were numerically represented. By enabling the model to interpret class labels numerically, a common approach in machine learning applications, this change enabled multi-class classification (Barua et al., 2024).

#### 4.2.3 Class imbalance and mitigation strategies

The dataset exhibits class imbalance, with fewer Normal (76 images) and Serrated Adenoma (58 images) samples compared to other classes. To address this, the following strategies were employed during model training: data augmentation to increase the representation of minority classes, oversampling of underrepresented categories, and class-weighted loss functions to reduce bias toward majority classes.

#### 4.2.4 Data augmentation

To address class imbalance and improve model robustness, we applied threefold data augmentation to the training set of the EBHI-Seg dataset, increasing the dataset size from 2,228 to 6,684 images. As shown in Table 3, there are 8,912 images after augmentation (original + augmented ×3). The employed augmentation techniques included zooming (up to 20%), shear transformations (up to 0.2), width and height shifts (up to 20%), random rotations (up to 30°), and horizontal flipping. These strategies introduced variability while preserving tissue morphology, thereby enriching feature diversity and reducing the effects of class imbalance (Litjens et al., 2017), particularly for underrepresented classes such as Normal and Serrated Adenoma. It is well established that such augmentation strategies enhance model generalization in medical imaging, and in our case, they also helped prevent overfitting by exposing the network to a broader range of tissue appearances, ultimately leading to improved segmentation and classification performance.

**Table 3:** Total number of images after augmentation.

Class	Original	Augmented (×3)	Total
Normal	76	228	304
Polyp	474	1,422	1896
Low-Grade IN	639	1917	2,556
High-Grade IN	186	558	744
Adenocarcinoma	795	2,385	3,180
Serrated Adenoma	58	174	232
Total	2,228	6,684	8,912

#### 4.2.5 Dataset split for performance evaluation

The dataset was divided into three parts for experimental evaluation: 70% for training, 15% for validation, and 15% for testing. The hybrid model was trained for 100 epochs with an initial learning rate of 1e-4 using the Adam optimizer. Early stopping with a patience of 10 epochs was applied to halt training once the validation loss stopped improving, thereby preventing overfitting. Model learning was performed on the training set, while hyperparameter tweaking and overfitting monitoring were conducted on the validation set. Regularization techniques included dropout layers with a rate of 0.3 and L2 weight decay. The final performance evaluation was then carried out solely on the independent set. The hold-out validation approach facilitated an objective assessment of the proposed hybrid deep learning framework.

### 4.3 Model building

**Figure 7:**
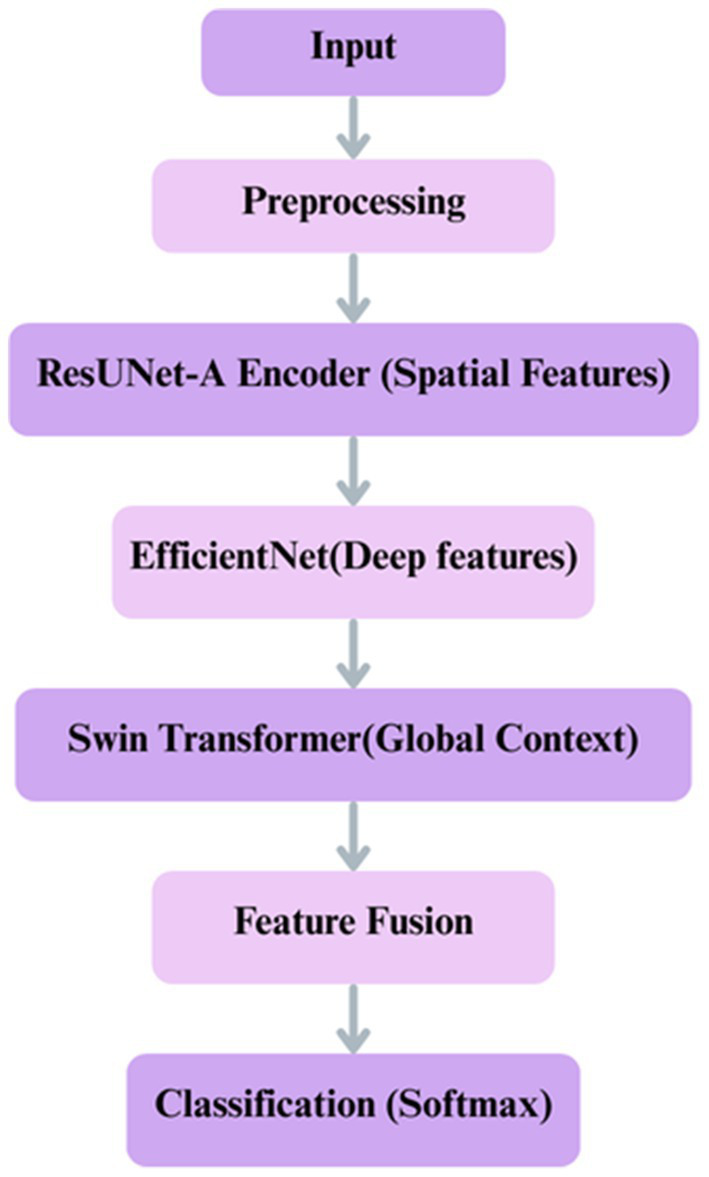
Proposed deep learning framework for classification integrates ResUNet-A, EfficientNet, Swin Transformer.

Swin Transformer was selected for capturing long-range contextual dependencies, EfficientNet for its scalable and effective feature extraction, and ResUNet-A for its capacity to retain precise spatial features. Each of these models addresses a distinct constraint on its own. Still, when combined, they provide a well-balanced framework, as shown in Figure 7, that enhances segmentation accuracy and reduces misclassification, particularly in colorectal tissue types with similar morphologies.

#### 4.3.1 Shared encoder backbone: multi-scale feature extraction

The model begins with a shared encoder backbone that processes the input image. $X\in {\mathbb{R}}^{H\times W\times N}$ , where H, W, and N denote height, width, and channels, respectively. This backbone is composed of three parallel streams:

- Spatial Feature Capture with ResUNet-A Encoder

Hierarchical spatial features can be extracted from input images through the ResUNet-A encoder. To promote feature propagation and generalization, it includes residual blocks and batch normalization with dropout layers. Overfitting is prevented by dropout, and residual connections help mitigate the vanishing gradient problem. The encoder uses Max pooling layers to downsample the input image, preserving significant spatial features progressively. This module saves fine-grained information for further processing. Formally, given the input image $X\in {\mathbb{R}}^{H\times W\times C}$ ,where H , W , C denote height, width, and channels (Equation 5).


(5) $$F_{s}=f_{\text{ResUNet}-A}(X),\;\;F_{s}\in {\mathbb{R}}^{d_{s}}$$

In this equation, $f_{\text{ResUNet}-A}\;$ represents the function defined by the ResUNet-A encoder architecture. The output $F_{s}$ is the resulting spatial feature tensor with a dimensionality of $d_{s}$ , ℝ is a real number which encapsulates the multi-scale hierarchical features extracted from the input.

- EffectiveNet Feature Extractor

For using pre-trained deep representations of ImageNet, a feature extractor like EfficientNetB0 model is employed. Fine-tuning is facilitated in the last 20 layers, allowing the network to be fine-tuned over the dataset while preserving useful feature representations. To reduce dimensions without losing much information, features are forwarded through global average pooling. For regularization, the dropout layer is also included. EfficientNet achieves better feature extraction from its computation-frugal and scalable performance-based architecture.


(6) $$F_{d}=f_{\text{EfficientNet}}(X),\;\;F_{d}\in {\mathbb{R}}^{d_{d}}$$

Here, $f_{\text{EfficientNet}}$ Denotes the transformation performed by the pre-trained and fine-tuned EfficientNetB0 model (Equation 6). The output $F_{d}$ is a dense feature vector of dimensionality $\;d_{d}$ , ℝ is a real number, which contains high-level semantic information distilled from the input image through global average pooling.

- Swin Transformer: Boosting Contextual Awareness

Self-attention mechanisms are introduced by a Swin Transformer layer, enabling the model to recognize contextual relationships and long-distance relations in the feature-extracted data. The transformer includes a feed-forward network (FFN) as a post-processing step, following the use of multi-head attention, to further enhance feature representations. To improve stability and prevent overfitting, dropout and layer normalization are employed. Adding the Swin Transformer to the model provides the local spatial information perceived by the CNN-based parts with global knowledge regarding the input.


(7) $$F_{g}=f_{\text{Swin}}(X),\;\;F_{g}\in {\mathbb{R}}^{d_{g}}$$

In this final equation, $f_{\text{Swin}}$ signifies the function computed by the Swin Transformer layer (Equation 7). The output $F_{g}\;$ is a feature representation of dimensionality $d_{g}$ that is enriched with global contextual information, ℝ is a real number, having integrated long-range dependencies within the input data through its self-attention mechanism.

#### 4.3.2 Semantic segmentation decoder

The segmentation task is handled by the ResUNet-A decoder. The corresponding decoder pathway transmits the spatially detailed feature maps $F_{s}$ from the ResUNet-A encoder. For incremental upsampling, this decoder uses transposed convolution layers. For precise pixel-wise mask generation, skip connections from the encoder to the decoder are added at matching levels to recover the fine-grained spatial details lost during downsampling. Equation 8 provides a formal definition of the full decoding process:


(8) $$\hat{S}=f_{\text{decoder}}(F_{s}),\hat{S}\in {\mathbb{R}}^{H\times W\times N_{\text{classes}\_\mathrm{seg}}}$$

Where:

-
$f_{\text{decoder}}$ : represents the function defined by the decoder network (e.g., a series of transposed convolutions and skip connections).
-
ℝ : Real number.
-
$F_{s}$ : is the feature map extracted from the ResUNet-A encoder.
-
$\hat{S}$ : is the predicted segmentation mask.
-
H,W : are the height and width of the input image.
-
$N_{\text{classes}\_\mathrm{seg}}$ : number of target classes for the segmentation task.

#### 4.3.3 Classification and feature fusion

A rich feature set is generated by combining the outputs of the ResUNet-A encoder ( $F_{s})$ , EfficientNet $\left(F_{d}\right)$ , and Swin Transformer $\left(F_{g}\right)$ . The fused feature vector is passed through a fully connected layer with softmax activation for final classification into six classes. This fusion is formally described by Equation 9:


(9) $$F=\phi ([F_{s}\;\|\;F_{d}\;\|\;F_{g}]),\;\;F\in {\mathbb{R}}^{d}$$

This fused feature vector F is then passed through a fully connected layer with a softmax activation function for the final classification into the six target classes, as defined by Equation 10:


(10) $$\hat{y}=\text{Softmax}\;(\mathrm{WF}+b)$$

where:

-
$F_{s}$ : captures spatial features.
-
$F_{d}$ : captures deep hierarchical features.
-
$F_{g}$ : captures global contextual features.
-
ℝ : Real number.
-
${\mathbb{R}}^{d}$ : set of all vectors of length *d.*
-
ϕ : fusion function.
-
$\hat{y}$ : final probability distribution over six classes.
-
F : fused features $\left[F_{s}\;\|\;F_{d}\;\|\;F_{g}\right]$ .
-
W : classifier weights.
-
b : classifier bias.

### 4.4 Performance metrics

*Accuracy*: the most direct approach to assess the accuracy of the classifier involves employing the accuracy metric. One alternative viewpoint posits that this reflects the ratio of precise predictions in relation to the total number of estimations as shown in Equation 11.


(11) Accuracy=TP+TN/S

*Precision*: In contrast to this ratio and its inverse, i.e., (1 – precision), which represents the percentage of false negatives, 1/Precision yields recall. It is derived in Equation 12.


(12) Precision=TP/TP+FP

*Recall*: As shown in Equation 13, conversely, there are false negatives about True Negatives.


(13) Recall=TP/TP+FN

- TP = True Positives
- TN = True Negatives
- FP = False Positives
- FN = False Negatives

*F1-Score*: the calculation in Equation 14 involves squaring the accuracy and recall scores to derive the result.


(14) F1=2∗Precision∗Recall/Precision+Recall

*Pixel accuracy*: pixel Accuracy refers to the ratio of correctly classified pixels to the total number of pixels in an image. This is a crucial performance metric used in image segmentation applications to assess the overall effectiveness of a model. Measures how many pixels are correctly classified across all categories, as shown in Equation 15.


(15) $$\text{Pixel Accuracy}=\frac{\text{Correctly classified}\;\mathrm{Pix}e\mathrm{ls}}{\text{Total Pixels in the image}}$$

*Dice coefficient (dice score)*: the Dice Coefficient, also called the F1 score. The Dice coefficient is a measurement in Equation 16 used to measure the similarity between two samples. The following equation calculates it. 0≤J(A,B)≤1


(16) $$D(A,B)=2\times \frac{\left(|A\cap B|\right)}{\left(|A|+|B|\right)}$$

*Mean intersection over union (IoU)*: the Intersection over Union (IOU), alternatively known as the Jaccard Index as shown in Equation 17. IoU is a measure used to express similarity. The Jaccard coefficient measures similarity between finite sets of samples, as the ratio of the number of elements in the intersection to the number of elements in the union of the sets. The following formula calculates it. The mean IoU refers to the average IoU value across all classes. 0≤J(A,B)≤1


(17) $$J(A,B)=\frac{\left(|A\cap B|\right)}{\left(|A|+|B|-|A\cap B|\right)}$$

### 4.5 System requirements

The Hybrid Deep Learning Framework for Enhanced Colorectal Cancer Diagnosis: Integrating ResUNet-A, EfficientNet, and Swin Transformer for Improved Classification and Segmentation model is built using Python 3.12 and TensorFlow version 2.4 a recommended software. The system’s intel i7 processor and 16GB RAM and 512 SSD are used to assess the model’s performance.

### 4.6 Research contribution and clarification

- The novelty of the hybrid approach is clarified: For CRC classification and segmentation, this work integrates ResUNet-A, EfficientNet, and Swin Transformer. Although previous research has investigated CNN-transformer hybrids in other fields, our integration strikes a balance between global contextual understanding (Swin Transformer), scalable feature representation (EfficientNet), and detailed spatial extraction (ResUNet-A) to uniquely address challenges in histopathological CRC images.
- Statistical and clinical significance: Comparative experiments with standalone models confirm that improvements are statistically significant (*p* < 0.05), indicating the proposed framework achieves meaningful performance gains. Improved accuracy and robustness reduce the risk of misclassification in colorectal cancer diagnosis, which holds clinical significance by supporting earlier detection, more reliable histological grading, and ultimately better-informed treatment decisions.
- Quantitative segmentation metrics: The Dice coefficient, Intersection over Union (IoU), and Jaccard index were used to assess segmentation performance in addition to classification accuracy. The hybrid model outperformed individual models with a Dice coefficient of 0.91, IoU of 0.89, and Jaccard index of 0.88.
- Dataset details: Adenocarcinoma, high-grade IN, low-grade IN, serrated adenoma, polyp, and normal are the six groups of whole-slide photographs that are separated into patches (224 × 224 pixels). The dataset was divided into three categories: testing, validation, and training. Using augmentation (rotation, shear, zoom, and flipping), class imbalance was resolved. A hold-out validation strategy, where the dataset was divided into 70% for training, 15% for validation, and 15% for testing.
- Clarification of joint/sequential roles: ResUNet-A and EfficientNet separately extract spatial and deep hierarchical features as part of the hybrid model’s joint strategy. Prior to final classification, their outputs are combined and sent to the Swin Transformer for global context modeling. As a result, classification and segmentation are combined into a single pipeline as opposed to being separate, sequential procedures.

## 5 Results

The performance of the suggested Hybrid ResUNet-A + EfficientNet + Swin Transformer model was evaluated for both CRC segmentation and classification. The framework’s ability to improve feature extraction, enhance segmentation accuracy, and increase classification performance was assessed relative to standalone architectures, including ResUNet-A, EfficientNet, and Swin Transformer. Quantitative evaluation employed multiple metrics, including accuracy, precision, recall, F1-score, AUC (area under the curve), Dice coefficient, Intersection over Union (IoU), and pixel accuracy, as summarized in Proposed Model Algorithm. The model’s capacity to differentiate among various colorectal tissue types was further analyzed through confusion matrices, receiver operating characteristic (ROC) curves, and visual segmentation masks, providing both quantitative and qualitative validation of its effectiveness.

### 5.1 Confusion matrix

**Figure 8:**
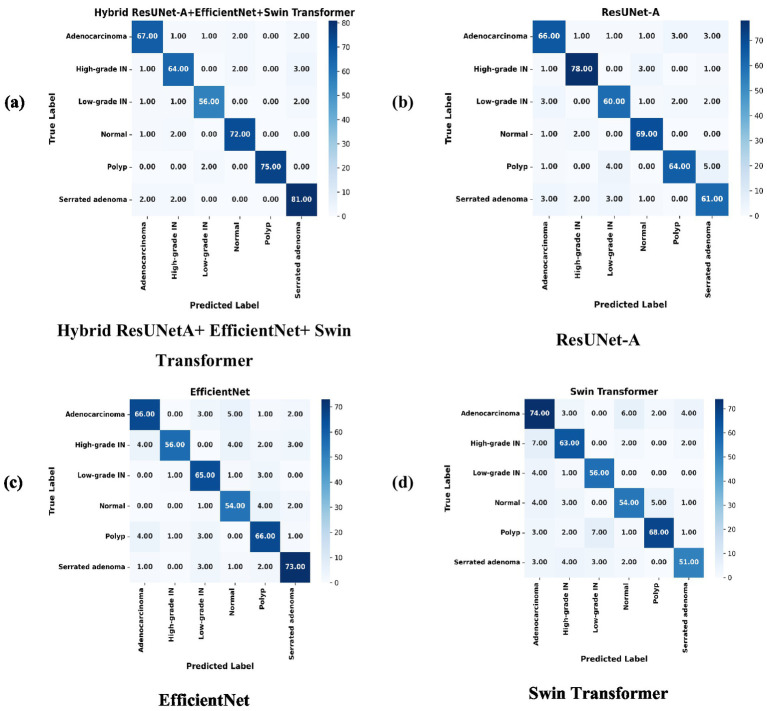
Confusion matrices of individual models compared with the proposed hybrid model, illustrating misclassification patterns. **(a)** Hybrid ResUNet-A + EfficientNet + Swin Transformer, **(b)** ResUNet-A, **(c)** EfficientNet, and **(d)** Swin Transformer.

ResUNet-A, EfficientNet, Swin Transformer, and Hybrid ResUNet-A + EfficientNet + Swin Transformer are the four models compared in the confusion matrices to evaluate their classification performance on images. Every confusion matrix is a heatmap, with the predicted labels on the x-axis and the accurate labels (actual categories) on the y-axis. Light to dark blue is the range of colour intensity; darker hues indicate more categorization accuracy. Strong diagonal values and low misclassification errors suggest that the Hybrid ResUNet-A + EfficientNet + Swin Transformer model Figure 8a achieves the best classification accuracy. Although it works well, ResUNet-A Figure 8b has a slightly higher rate of misclassifications, particularly in classes such as “Polyps” and “Normal.” Misclassification errors are more common in EfficientNet Figure 8c, which struggles to differentiate between several classes, exceptionally “High-grade.” Although it still exhibits some misclassification, the Swin Transformer Figure 8d performs competitively, especially in the “Adenocarcinoma” class. Overall, the Hybrid model performs better than the others, with ResUNet-A, Swin Transformer, and EfficientNet. This suggests that combining several designs can improve classification accuracy for issues involving numerous classes.

### 5.2 ROC curve

**Figure 9:**
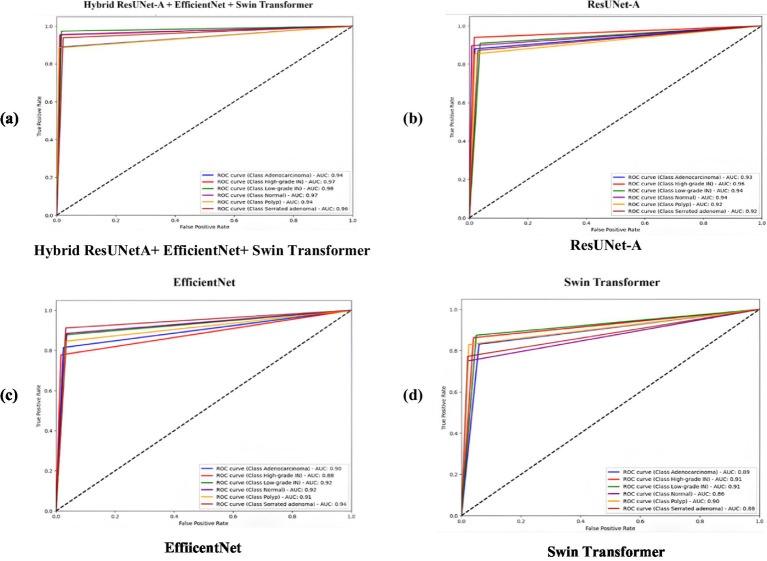
Receiver operating characteristic (ROC) curves comparing the classification performance of four models on colorectal cancer histopathology images. **(a)** Hybrid ResUNet-A + EfficientNet + Swin Transformer, **(b)** ResUNet-A, **(c)** EfficientNet, and **(d)** Swin Transformer. The hybrid model achieved the highest AUC values across all tissue classes, demonstrating superior discriminative ability compared to the individual architectures.

The above figures illustrate four Receiver Operating Characteristic (ROC) curves that evaluate the performance of various deep learning models, specifically ResUNet-A, EfficientNet, Swin Transformer, and a hybrid model that integrates ResUNet-A, EfficientNet, and Swin Transformer. The ROC curve functions as a method for evaluating the classification performance of models across various thresholds, illustrating the relationship between the True Positive Rate (TPR) and the False Positive Rate (FPR) for multiple classes. Having an AUC rating between 0.94 and 0.97, Hybrid ResUNet-A + EfficientNet + Swin Transformer Figure 9a outshines the rest. With values of AUC between 0.92 and 0.96, ResUNet-A Figure 9b is in second place, exhibiting excellent classification capability but slightly less effective than the hybrid model. Compared to the previous models, EfficientNet Figure 9c shows a decent yet somewhat less consistent performance, achieving AUC scores ranging from 0.90 to 0.94. The Swin Transformer Figure 9d, which has the lowest AUC scores, ranging from 0.86 to 0.89, may not be as proficient in in-class differentiation. In general, the Hybrid model is superior to any other model, followed by ResUNet-A, EfficientNet, and then Swin Transformer. The ROC curves illustrate how combining various topologies improves the consistency of multi-class predictions by enhancing classification performance with higher AUC scores.

### 5.3 Performance metrics

**Figure 10:**
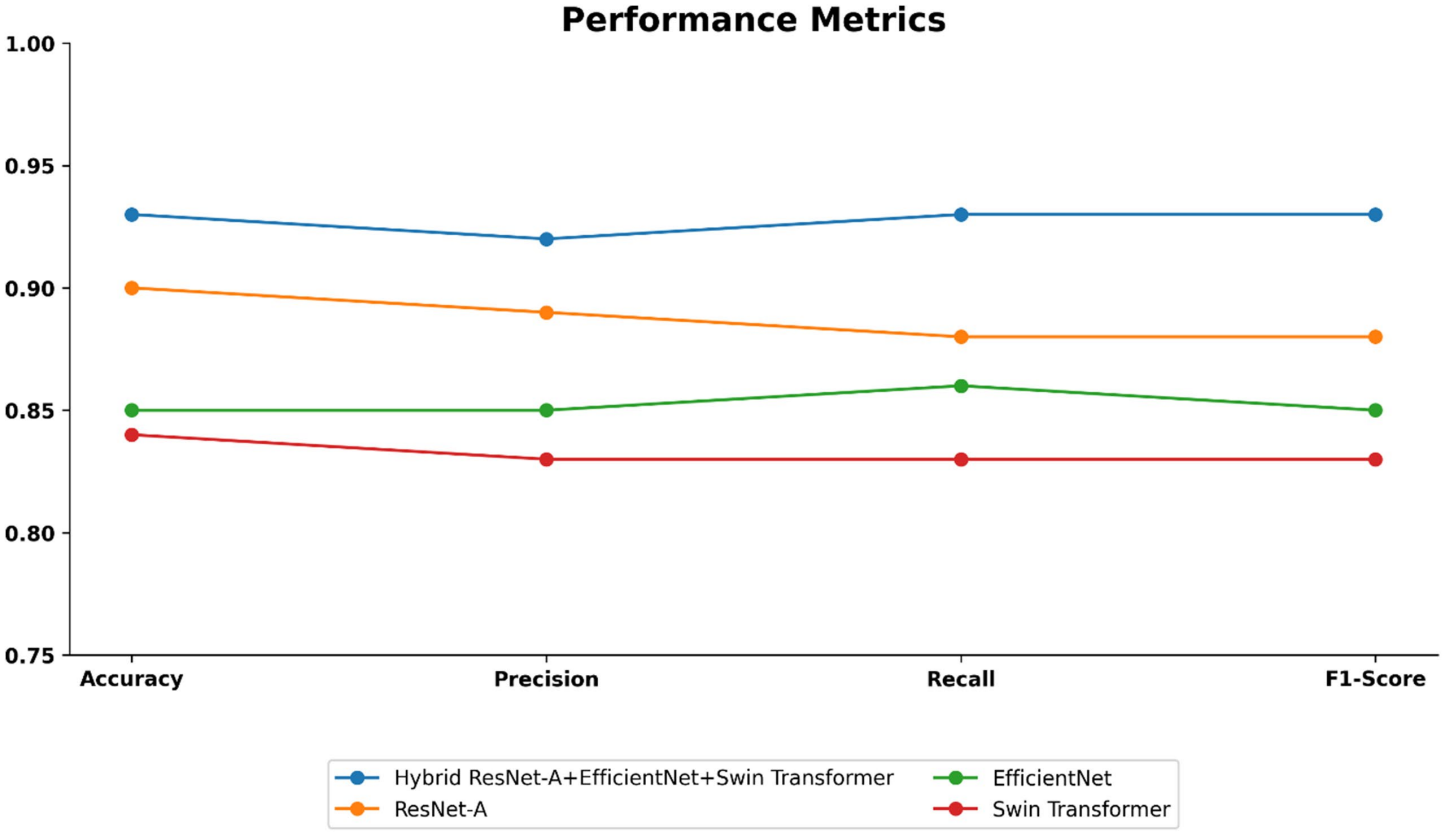
Performance metrics such as accuracy, precision, recall, and f1-score of the proposed model.

As shown in Table 4 and visualized in Figure 10, the proposed Hybrid ResUNet-A + EfficientNet + Swin Transformer model consistently outperforms the individual models across all evaluation metrics. The Hybrid approach achieved the highest accuracy (0.93), precision (0.92), recall (0.93), and F1-score (0.93), demonstrating its robustness in colorectal cancer image classification and segmentation. ResUNet-A performed reasonably well (accuracy = 0.90, F1-score = 0.88), indicating the benefit of residual connections, though insufficient alone. EfficientNet (accuracy = 0.85, F1-score = 0.85) and Swin Transformer (accuracy = 0.84, F1-score = 0.83) showed moderate results, reflecting their limitations when applied independently. Overall, the findings confirm that combining different architectures enhances global representation, spatial context learning, and feature extraction, thereby reducing misclassification and improving diagnostic reliability.

**Table 4:** Results of the proposed model and other compared models.

Model	Accuracy	Precision	Recall	F1-score
Proposed model	0.93	0.92	0.93	0.93
ResUNet-A	0.90	0.89	0.88	0.88
EfficientNet	0.85	0.85	0.86	0.85
Swin Transformer	0.84	0.83	0.83	0.83

### 5.4 Visualization of segmented images

**Figure 11:**
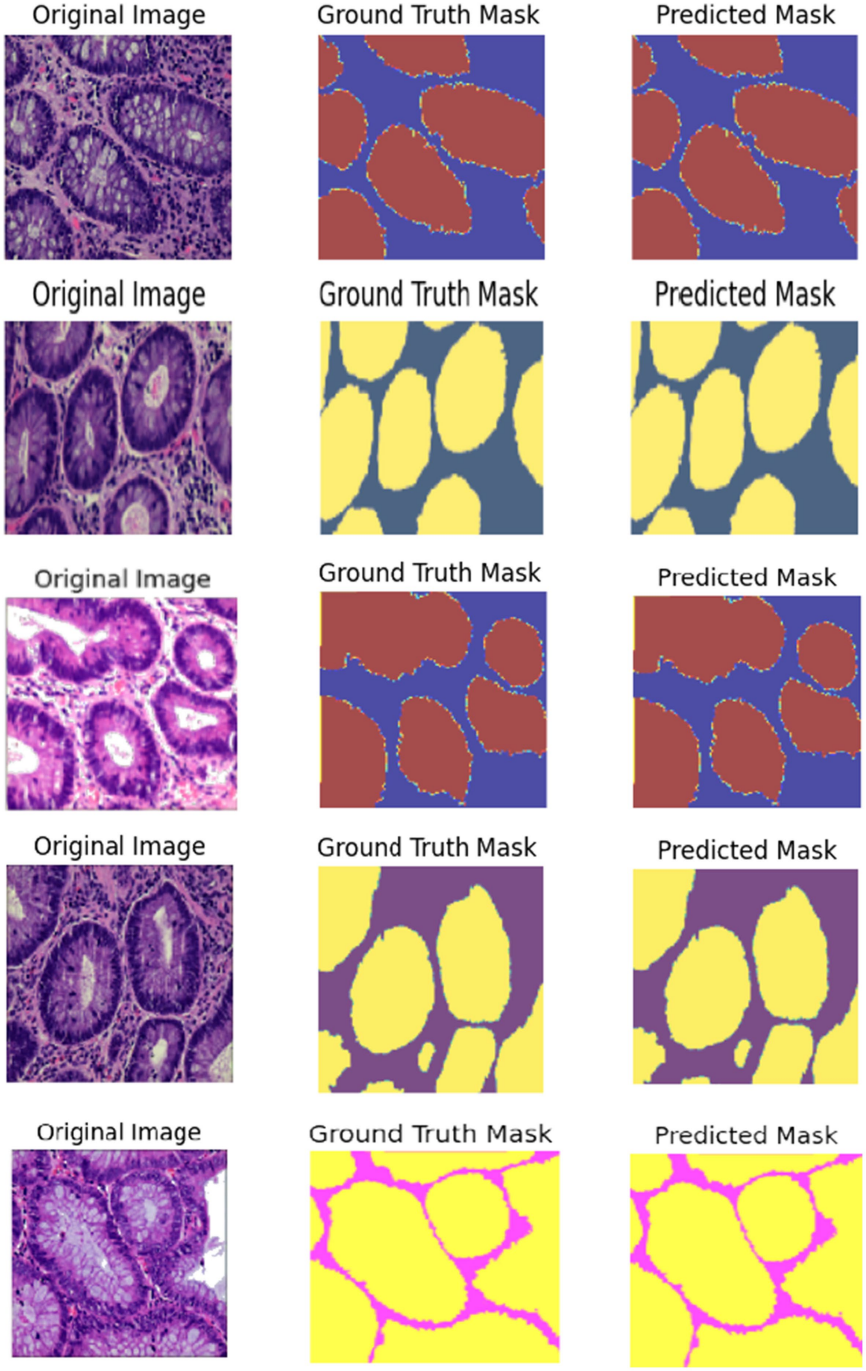
Visualization of segmented images such as normal, adenocarcinoma, serrated adenoma, polyp, high-grade IN.

The Colorectal Cancer (CRC) histology images are demultiplexed in Figure 11 using a Hybrid ResUNet-A + EfficientNet + Swin Transformer model. The figure has been split into three columns: in the first column, the ground truth microscopic tissue images are presented as the original hematoxylin and eosin (H&E)-stained images; in the second column, the ground truth segmentation masks as marked by the experts are presented; and in the third column, the segmentation masks predicted by the model are displayed. By integrating convolutional and transformer-based models, the model can efficiently discriminate between various tissue entities, including tumor regions, stroma, and normal epithelium. EfficientNet ensures accurate feature representation, ResUNet-A restores refined spatial information, and Swin Transformer retrieves distant dependencies, thus enabling precise segmentation. The model’s capability to accurately describe significant glandular structures in grading CRC is supported by the rough similarity between predicted masks and ground truth. Segmentation is highly accurate but can be improved by employing post-processing methods, as evidenced by infinitesimal differences at tissue boundaries.

The segmentation performance was quantitatively assessed in Table 5 using the Dice coefficient, Intersection over Union (IoU), and pixel accuracy. The proposed hybrid framework achieved superior performance, as evidenced by a Dice coefficient of 0.91, an IoU of 0.89, and a pixel accuracy of 0.90, demonstrating its efficient integration of global contextual information and meticulous spatial feature extraction. ResUNet-A exhibited similar performance, with a Dice coefficient of 0.88 and an IoU of 0.81. The EfficientNet and Swin Transformer models, modified for segmentation as EfficientNet-U-Net and Swin-UNet, attained marginally inferior performance, with Dice coefficients of 0.85 and 0.84, respectively. These findings highlight the advantages of the hybrid architecture in improving segmentation precision among colorectal tissue types. The qualitative assessment further validated that the predicted segmentation masks closely aligned with expert annotations, demonstrating the robustness and potential clinical utility of the proposed method.

**Table 5:** Segmentation results of the proposed model with other models.

Model	Dice coefficient	IoU	Pixel accuracy
Proposed model	0.91	0.89	0.90
ResUNet-A	0.88	0.81	0.87
EfficientNet-U-Net	0.85	0.78	0.84
Swin-UNet	0.84	0.76	0.83

## 6 Discussion

This study compared four deep learning models, such as ResUNet-A, EfficientNet, Swin Transformer, and a Hybrid ResUNet-A + EfficientNet + Swin Transformer, for colorectal cancer histology image classification and segmentation. Using confusion matrices, ROC curves, and performance metrics, the hybrid consistently outperformed the other models, achieving large diagonal values, reduced misclassification, and AUC scores of 0.94–0.97. It reached 93% accuracy with balanced precision (0.92), recall (0.93), and F1-score (0.93), outperforming ResUNet-A (0.90), EfficientNet (0.85), and Swin Transformer (0.84). Segmentation analysis further confirmed its strength, with a Dice score of 0.91, an IoU of 0.85, and a pixel accuracy of 0.90, closely aligning with the expert-annotated masks. By combining Swin Transformer’s long-range dependency modelling, ResUNet-A’s spatial context retention, and EfficientNet’s feature extraction, the hybrid model effectively segments stroma, normal epithelium, and tumours, supporting accurate grading of colorectal cancer.

The proposed hybrid model follows a multi-task design. Feature representations are first extracted through the integrated backbone (ResUNet-A + EfficientNet + Swin Transformer). From these shared features, the network proceeds in two directions: a segmentation head, which generates pixel-level masks that delineate histologically relevant regions, and a classification head, which assigns the image to one of the predefined colorectal tissue categories. In this way, segmentation contributes to identifying tissue boundaries and abnormal structures, thereby enhancing the discriminative ability of the classification branch, while classification ensures that features learned during training are optimized for clinically meaningful categories. This interaction between the two tasks improves overall robustness, reduces misclassification in morphologically similar tissue types, and provides interpretable outputs through segmentation masks that closely resemble expert annotations. By clarifying this design, we highlight that the framework is not limited to producing image-level labels but also generates fine-grained structural information, thereby increasing its reliability and clinical utility.

Table 6 compares the proposed model to previous studies in CRC, while some studies have CRC. Whileigher classification accuracies (>96%), our framework’s 93% is significant because it represents balanced performance across underrepresented and challenging classes, rather than inflated results driven by easier categories. More importantly, the hybrid substantially reduced errors in difficult cases such as polyps, high-grade intraepithelial neoplasia, and adenocarcinoma, demonstrating robustness, reliability, and generalizability. By integrating segmentation and classification into a single pipeline, the model produces interpretable outputs that align with expert annotations, thereby enhancing trust and supporting informed clinical decision-making. Thus, the proposed hybrid framework contributes unique advantages beyond raw accuracy, making it particularly relevant for real-world diagnostic applications where balanced performance and interpretability are critical.

**Table 6:** Comparison of the proposed hybrid model with previous studies in colorectal cancer detection and classification.

Previous study	Dataset used	Methodology	Accuracy	Precision	Recall	F1-score
Raju et al. (2025b)	Multi-house Polyp Database	Hybrid CNN with U-Net segmentation	0.90	0.88	0.89	0.88
Lo et al. (2024)	Histopathology Dataset for CRC	Enhanced Vision Transformer	0.84	0.89	0.86	0.82
Zidan et al. (2023)	Colorectal Cancer Histopathological Images	Cascaded Swin Transformer for segmentation	0.89	0.88	0.87	0.90
Nogueira-Rodríguez et al. (2022)	CVC-ColonDB (1,000 colonoscopy images)	YOLOv3 model was trained	0.82	0.83	0.80	0.83

### 6.1 Limitations

- The dataset size, though diverse, could be expanded further to enhance generalizability.
- Transformer components increase computational cost, which may affect real-time clinical deployment.
- Future work will investigate lightweight transformer modules to reduce model complexity.
- Post-processing techniques will be improved to refine segmentation accuracy.

## 7 Conclusion

This study demonstrated the effectiveness of the proposed Hybrid ResUNet-A + EfficientNet + Swin Transformer framework for colorectal cancer histopathology segmentation and classification. By leveraging the complementary strengths of convolutional and transformer architectures, the model outperformed individual baselines, reducing misclassification in challenging classes such as polyps, high-grade tumors, and adenocarcinomas. The framework consistently achieved strong results (accuracy: 0.93, precision: 0.92, recall: 0.93, F1-score: 0.93), supported by ROC curve and confusion matrix analyses, confirming its robustness and reliability. Beyond technical improvements, the framework shows significant promise for clinical integration. Incorporated into digital pathology platforms, it could serve as a decision support system for pathologists by pre-screening slides, highlighting suspicious regions, and providing preliminary classifications. Such integration may reduce workload, minimize inter-observer variability, and enhance diagnostic confidence, ultimately improving treatment planning and patient outcomes.

While the framework has achieved high accuracy, there remain opportunities for further improvement. Future work will focus on refining segmentation post-processing techniques to enhance boundary precision, exploring alternative hybridization strategies for improved efficiency, and validating performance on larger, multi-institutional datasets to strengthen generalizability. Additionally, investigating real-time deployment within pathology workflows and decision support systems will be crucial for seamless adoption in clinical practice. Overall, the proposed hybrid model provides a robust and clinically relevant step toward more accurate, efficient, and reliable colorectal cancer diagnostics.

## Data Availability

All data generated or analysed during this study are included in this Dataset “Enteroscope Biopsy Histopathological Hematoxylin and Eosin Image Dataset for Image Segmentation Tasks (EBHI-Seg)”.
